# Prenatal Exposure to Bisphenol A at Environmentally Relevant Doses Adversely Affects the Murine Female Reproductive Tract Later in Life

**DOI:** 10.1289/ehp.0800045

**Published:** 2009-01-15

**Authors:** Retha R. Newbold, Wendy N. Jefferson, Elizabeth Padilla-Banks

**Affiliations:** Developmental Endocrinology and Endocrine Disruptor Section, Laboratory of Molecular Toxicology, National Institute of Environmental Health Sciences, National Institutes of Health, Department of Health and Human Services, Research Triangle Park, North Carolina, USA

**Keywords:** BPA, carcinogenesis, DES, development, diethylstilbestrol, endocrine disruptors, ovary, reproduction, toxicology, uterus

## Abstract

**Background:**

Exposure to endocrine-disrupting chemicals during critical developmental periods causes adverse consequences later in life; an example is prenatal exposure to the pharmaceutical diethylstilbestrol (DES). Bisphenol A (BPA), an environmental estrogen used in the synthesis of plastics, is of concern because its chemical structure resembles that of DES, and it is a “high-volume production” chemical with widespread human exposure.

**Objectives:**

In this study we investigated whether prenatal BPA causes long-term adverse effects in female reproductive tissues in an experimental animal model previously shown useful in studying effects of prenatal DES.

**Methods:**

Timed pregnant CD-1 mice were treated on days 9–16 of gestation with BPA (0.1, 1, 10, 100, or 1,000 μg/kg/day). After delivery, pups were held for 18 months; reproductive tissues were then evaluated.

**Results:**

Ovarian cysts were significantly increased in the 1-μg/kg BPA group; ovarian cyst-adenomas were seen in the other three BPA-treated groups but not in corn-oil controls. We observed increased progressive proliferative lesions of the oviduct after BPA treatment, similar to those described in response to DES. Further, although not statistically different from the controls, prominent mesonephric (Wolffian) remnants and squamous metaplasia of the uterus, as well as vaginal adenosis, were present in BPA-treated mice, similar to lesions reported following DES treatment. More severe pathologies observed in some BPA-treated animals included atypical hyperplasia and stromal polyps of the uterus; sarcoma of the uterine cervix; and mammary adenocarcinoma. We did not observe these lesions in controls.

**Conclusions:**

These data suggest that BPA causes long-term adverse reproductive and carcinogenic effects if exposure occurs during critical periods of differentiation.

Perinatal exposure to environmental chemicals, in particular those with estrogenic activity, can have long lasting consequences if exposure occurs during critical periods of reproductive tract development (Bern 1992; Marselos and Tomatis 1992a, 1992b; McLachlan and Newbold 1996; Newbold and McLachlan 1996; Tomatis et al. 1992; Yamasaki et al. 1992). More recent studies have further proposed that hormonal perturbations during fetal or neonatal development may predispose individuals to numerous diseases and/or dysfunction later in life, such as hypertension and coronary disease (Sallout and Walker 2003); obesity (Heindel 2003; Newbold et al. 2005, 2007; Oken and Gillman 2003); reproductive problems including infertility/subfertility (Newbold 2004); and increased tumors such as uterine fibroids (leiomyomas) (Newbold et al. 2000) and breast cancer (Davis et al. 1993). This phenome non is referred to as “the developmental origins of health and disease” (Gluckman et al. 2005). The benign and carcinogenic effects of prenatal exposure to diethylstilbestrol (DES), a potent synthetic estrogen used to prevent miscarriage in the late 1940s through the 1970s, is an example of the harmful effects that estrogenic chemicals can cause during development, many of which are not apparent until much later in adult life. Although the use of DES during pregnancy was discontinued > 30 years ago, we are still reminded of this chemical’s legacy as the DES-exposed offspring age and their health care problems continue to mount [National Institutes of Health (NIH) 1999; Palmlund 1996] including the possibility of adverse effects on subsequent generations (Blatt et al. 2003; Newbold 2004; Titus-Ernstoff et al. 2008). Unfortunately, developing fetuses and young children continue to be inadvertently exposed to a wide number of environmental chemicals, many with hormone-like activity. Some of these chemicals occur in high production volumes, and exposure comes from a number of different sources.

Bisphenol A (BPA), a monomer component of polycarbonate plastics and epoxy resins, is one such hormonally active chemical that is receiving increased attention due to its high production volume and widespread human exposure. BPA is used in the manufacture of numerous products and has been shown to leach from the linings of food cans (Brotons et al. 1994), polycarbonate baby bottles and other beverage containers (Biles et al. 1997), dental sealants and composites (Olea et al. 1996), and polyvinylchloride plastics and recycled thermal paper (European Communities 2008), suggesting that humans are routinely exposed to this chemical through numerous sources and routes of exposure. Further indication of human exposure is shown by studies reporting measurable BPA levels in human urine (Calafat et al. 2008), serum (Takeuchi and Tsutsumi 2002), breast milk (Ye et al. 2006), and maternal and fetal plasma, amniotic fluid, and placental tissues (Padmanabhan et al. 2008; Schonfelder et al. 2002).

BPA is most commonly described as being a “weak” estrogen; however, an emerging number of cellular and molecular studies show that it has potential for many other biological activities at environmentally relevant exposures. In addition to binding to the nuclear estrogen receptors ER-α and ER-β, BPA interacts with a variety of other cellular targets including binding to a nonclassical membrane-bound form of the ER (ncmER), a recently identified orphan nuclear receptor termed estrogen-related receptor-γ (ERRγ), a seven-transmembrane estrogen receptor called GPR30, and the aryl hydrocarbon receptor (AhR). Interactions with ncmER and ERRγ are especially noteworthy because BPA binds to these receptors with high affinity. BPA also has been shown to act as an androgen receptor antagonist and to interact with thyroid hormone receptors (for review, see Wetherill et al. 2007).

Studies in experimental animals have shown that very low doses of endocrine-disrupting chemicals (Melnick et al. 2002), in particular BPA, in the range of human exposures can exert effects if administered during development (Markey et al. 2005). An increasing number of “low-dose” studies have suggested that perinatal BPA exposure is associated a variety of abnormalities in the female reproductive tissues, including early onset of vaginal opening (Honma et al. 2002), early onset of puberty (Howdeshell et al. 1999; Ryan 2006), altered estrus cyclicity (Markey et al. 2003), altered plasma levels of luteinizing hormone (Rubin et al. 2001), altered vaginal and uterine histology (Schonfelder et al. 2002, 2004), alterations in the mammary gland (Markey et al. 2001) and uterus (Markey et al. 2005), and altered ovarian morphology (Markey et al. 2003). Of greater concern, recent studies have reported an association of perinatal BPA exposure with more severe pathologies including preneoplastic and neoplastic lesions in male prostate (Ho et al. 2006) and female mammary gland (Durando et al. 2007; Murray 2007). Although perinatal BPA exposure has been linked to early changes in the uterus, vagina, and ovary, no studies have examined the potential for low doses of BPA to cause potential carcinogenic alterations in these tissues. Low-dose exposure is particularly important because of its relevance to the levels experienced by the general human population (Calafat et al. 2008; Vandenberg et al. 2007).

The carcinogenic potential of BPA has been studied *in vitro*. High doses of BPA transform Syrian hamster embryo (SHE) cells and induce aneuploidy, suggesting that it has the potential to be genotoxic (Tsutsui et al. 1998, 2000). At doses close to human exposure levels, BPA has not been previously shown to be a classical mutagen, although it does cause meiotic aneuploidy in female germ cells grown in culture (Hunt et al. 2003). The National Toxicology Program (NTP) conducted *in vivo* carcinogenic studies of BPA in which the chemical was administered to groups of mature male and female rats and mice; the study concluded that “there was no convincing evidence that BPA was a carcinogen” (NTP 1982). However, there was a lack of consensus for the NTP conclusion (Huff 2003) because cancers of the hematopoietic system, increases in interstitial cell tumors of the rat testis, and mammary gland fibroadenomas were reported in male rats (NTP 1982). Further, the full carcinogenic potential of BPA was not determined because the NTP study did not include developmental exposure (Huff 2003).

The present study was designed to determine if prenatal exposure to BPA at low environmentally relevant doses causes long-term adverse effects in reproductive tissues of aged females. Based on reported data (Markey et al. 2005; vom Saal et al. 1997), we administered BPA to pregnant mice at 0.1–1,000 μg/kg maternal body weight on days 9–16 of gestation, estimating that these doses are relevant to human exposure (Calafat et al. 2008; Vandenberg et al. 2007). For example, estimated daily intakes of BPA in formula-fed infants range from 1 to 13 μg/kg (NTP 2008). We chose to administer BPA by subcutaneous (sc) injection because this route has proven successful in duplicating and predicting the effects of oral DES exposure in humans (Newbold 2004). Additional support for using sc injections was shown recently by Taylor (2008) who found no effect of route of exposure, either sc or oral, on plasma BPA levels in the neonatal female mouse. Also, in a study using adult mice, Alonso-Magdalena et al. (2006) showed that BPA treatment caused insulin resistance regardless of whether exposure was by injection or oral treatment, suggesting that the route of BPA exposure did not alleviate its effects.

## Materials and Methods

### Animals and treatment

Adult female CD-1 [Crl:CD-1 (ICR) BR] mice were obtained from Charles River Breeding Laboratories (Raleigh, NC) and bred to male mice of the same strain in the breeding facility at the National Institute of Environmental Health Sciences (NIEHS). Vaginal plug detection was considered day 0 of pregnancy. Pregnant mice were individually housed in polysulfone-ventilated cages (Technoplast, Inc.; Exton, PA) with hardwood chip bedding under controlled lighting (12 hr light/12 hr dark) and temperature (21–22°C) conditions. Mice were fed NIH 31 mouse chow, which was assayed for estrogenic activity as previously described (Thigpen et al. 1999), and fresh water was provided in polycarbonate water bottles *ad libitum.* All animal procedures complied with NIEHS/NIH animal care guidelines. All animals were treated humanely and with regard for alleviation of suffering.

Thirty timed pregnant mice (five per treatment group) were injected sc with corn oil (control) or BPA (> 99% purity; Sigma-Aldrich, Inc., St. Louis, MO) dissolved in corn oil on days 9 through 16 of gestation, the period of major organogenesis in the murine reproductive tract. The daily dose of BPA was 0.1, 1, 10, 100, or 1,000 μg/kg/day; we refer to the offspring of these animals as BPA-0.1, BPA-1, BPA-10, BPA-100, and BPA-1000, respectively. Pregnant mice delivered their young on day 19 of gestation. We observed no significant difference in litter size between control and BPA-treated mice. To minimize any potential prenatal litter effects, all pups in a treatment group were pooled together, separated by sex, and then fostered (four female and four male pups per litter) to moms of the same treatment group. Remaining male and female offspring were fostered to untreated moms and used in other experiments. At 21 days of age, offspring were weaned, separated by sex, housed four per cage, and held without further treatment. All mice were observed daily for sick and moribund appearance. Table 1 summarizes the number and disposition of female offspring; results for males will be described in a separate report.

Mice were euthanized by carbon dioxide asphyxiation at 16–18 months of age, or earlier if they were identified as being sick or moribund. Reproductive tract tissues including ovaries and oviducts were removed, fixed in 10% neutral buffered formalin, embedded in paraffin, and sectioned at 6 μm. Tissue sections were stained with hematoxylin and eosin (H&E) and evaluated by light microscopy. A minimum of five serial sections of reproductive tract tissues and nine ovary/oviduct sections were evaluated for each mouse. If a microscopic lesion was observed, additional serial sections were made to include the entire area of pathological change. In some cases, lesions were stained with Trichrome staining.

### Statistical analysis

We used Cochran-Armitage trend tests to evaluate dose-related changes in lesion incidence. We compared lesion incidence in each dose group with that in the control group using one-sided Fisher’s exact tests; *p*-values < 0.05 were considered statistically significant.

## Results

Although we started with 16 mice per treatment group, not all of the mice could be followed until 16–18 months of age. However, 84 of the 96 female mice were included. Table 1 shows the number of animals available at the end of the study and the frequency of sick and moribund animals. The BPA groups had more losses than the control group.

### Ovary/oviduct

**Figure 1:**
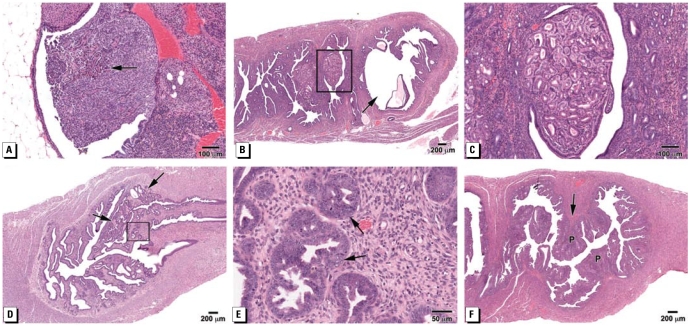
Photomicrographs of representative examples of abnormalities (H&E-stained tissue sections) in mice prenatally treated with BPA. (*A*) Cystadenoma of the ovary (BPA-100) composed of anastomosing papillary fronds (arrow). (*B*) Adenomatous hyperplasia and CEH (arrow) in the uterus (BPA-1), characterized by hyperplastic endometrial glands and foci of cystic dilated glands lined by flattened epithelium; some secretory material is present in the lumen of some glands. (*C*) Higher magnification of (*B*) showing hyperplastic endometrial glands lined by cells with regularly shaped nuclei with little to no cellular pleomorphism or mitotic activity. (*D*) Atypical hyperplasia in the uterus (BPA-0.1) composed of foci of irregularly shaped glands with little intervening stroma (arrows). (*E*) Higher magnification of (*D*) shows “piling up” of cells (arrows) with hyperchromatic nuclei and many mitotic figures. (*F*) Endometrial polyps (P) extending into the uterine lumen (BPA-1).

A comparison of ovarian and oviductal abnormalities in control and BPA-treated mice is shown in Table 2. In mice 16–18 months of age, we found no statistical difference in the number of mice without corpora lutea (CL) in any group. Cystic ovaries were also common in all groups, but only the BPA-1 group was significantly different from controls (*p* = 0.05). Prominent paraovarian cysts of mesonephric origin were present in one BPA-10 mouse [7% (1/14)]. Further, neoplastic lesions in the ovary included cyst-adenomas (Figure 1A), which were present in the BPA-10, BPA-100, and BPA-1000 groups but not in controls. These lesions were lined by nonciliated cubodial to columnar epithelial cells that were relatively uniform with basally located round nuclei.

We observed progressive proliferative lesion (PPL) of the oviduct in all groups of BPA-treated mice but not in controls. This oviductal abnormality has been previously described in prenatally DES-treated mice (Newbold et al. 1985).

### Uterus

The range of uterine abnormalities is also shown in Table 2. We observed cystic endometrial hyperplasia (CEH), a typical age-related change in mice, in all groups (control, 13%; BPA-1, 38%; BPA-10, 7%; BPA-100, 36%; and BPA-1000, 8%) except the BPA-0.1 group. Adenomyosis, characterized by benign invasion of endometrial glands into the myometrium, occurred in the BPA-0.1, BPA-10, and control groups; adenomyosis has been reported to be prevalent in DES-treated mice (McLachlan et al. 1980; Newbold et al. 1990). Unlike DES-treated animals, the BPA-treated and control mice in the present study had a well-developed uterine muscle wall; thus this lesion was different from DES-induced changes. Because of this difference and because the lesion was also present in controls, we did not consider it to be treatment related. However, adenomatous hyperplasia with CEH was seen in the BPA-1 and BPA-100 groups (Figure 1B, C) but not controls. Further, the more severe lesion of atypical hyperplasia of the uterus (Figure 1D, E), which is considered a precursor lesion to estrogen-associated uterine adenocarcinoma, occurred in 21% of the BPA-0.1, 15% of BPA-1, and 8% BPA-1000 animals, but not in any controls in the present study or in historical controls. We observed prominent Wolffian (mesonephric) remnants in the uterus similar to those seen in the ovary and oviduct in all BPA groups except BPA-100. Squamous metaplasia was also present in the BPA-1 and 100 groups.

**Figure 2:**
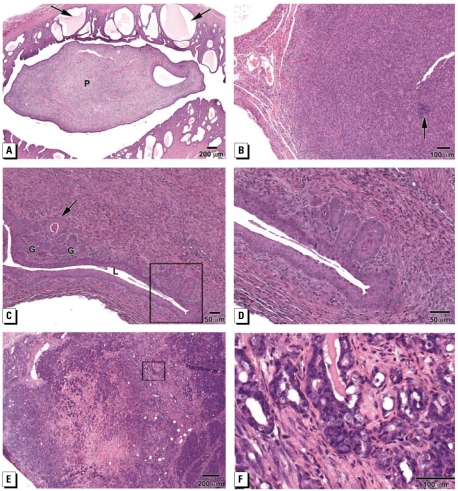
Photomicrographs of abnormalities in H&E-stained tissue sections from mice prenatally treated with BPA. (*A*) Endometrial stromal polyp (P) extending into the uterine lumen (BPA-10); note areas of CEH (arrow) with secretory material in glands. (*B*) Endometrial stromal sarcoma that has infiltrated into the uterine cervix (BPA-100). The lesion is composed of cells with pleomorphic nuclei with a moderate amount of mitotic activity; foci of inflammatory cells are indicated by the arrow. (*C*) Vaginal adenosis in tissue (BPA-1000) shows foci of “glandular structures” (G) lined by simple cuboidal to columnar epithelium. In one area, these “glands” were lined by squamous epithelium with keratinizing cells in the lumen (arrow); some of these glandular structures connected with the vaginal lumen (L), which is lined by stratified squamous epithelium. (*D*) Higher magnification of of vaginal adenosis in (*C*) showing glandular structures that connect to the squamous epithelium lining the vaginal lumen. (*E*) Mammary adenocarcinoma (BPA-1000) is a large solid tumor that has invaded the entire mammary structure and underlying muscle. (*F*) Higher magnification of (*E*) showing mammary adenocarcinoma composed of irregular shaped glands and pleomorphic cells with hyperchromatic nuclei.

Other lesions in the uterus included endometrial polyps, which were seen in the BPA-0.1, BPA-1 (Figure 1F), and BPA-10 groups. These polyps were pedunculated masses with well-organized stromal and smooth muscle components. Figure 2A shows a large stromal polyp (BPA-10), which extends into the uterine lumen and has areas of CEH with secretory material; these stromal lesions have been reported to be associated with the development of stromal cell sarcomas in rodents (Davis et al. 1999; Maronpot 1999). In fact, one BPA-100 mouse had a stromal sarcoma that had infiltrated into the uterine cervix (Figure 2B).

Although sections through the vaginal fornix were not always available, one BPA-1000 mouse had vaginal adenosis (Figure 2C, D) characterized by the abnormal location of “glandular structures” in the vagina. These glandular structures were lined by simple cuboidal to columnar epithelium. In one area, these cells had been replaced by squamous epithelium, and keratinizing cells were present in the glandular lumen (Figure 2C, D). Some of the glandular structures were connected to the vaginal lumen. Adenosis was reported in mice after prenatal or neonatal DES treatment (Newbold and McLachlan 1982), and it was the hallmark lesion seen in DES-exposed women (Herbst 1981).

In addition to mice that were sacrificed at the termination of the study, several mice became sick or moribund and were sacrificed early. One BPA-1 mouse, sacrificed at 7 months of age, had a poorly differentiated sarcoma that had invaded most of the reproductive tract, ovaries, and all major organs; this lesion was probably hematopoetic in origin and not primary to the reproductive tract. A BPA-1000 mouse was sacrificed at 10 months of age because of a large mammary mass, which was diagnosed as adenocarcinoma (Figure 2E, F). Another similar tumor was found in a BPA-1 mouse at necropsy at 18 months of age. We did not routinely screen the mammary gland, but these tumors were identified on gross examination at necropsy.

The total number of mice with lesions shown in Table 3 suggests that, overall, BPA is associated with increased tumor incidence of reproductive tissues if exposure occurs during prenatal life. The lowest dose tested, BPA-0.1, demonstrates the highest tumor incidence (36% of mice).

## Discussion

This article is the first to describe the induction of numerous abnormalities, including both benign and malignant lesions, in reproductive tissues of aged female mice exposed prenatally to a broad range of BPA doses (0.1–1,000 μg/kg maternal body weight). The profile of reproductive tract lesions observed in this study is similar to what we observed following neonatal treatment with BPA (Newbold et al. 2007). The BPA doses were low and within the range of human exposure, comparable with 1–13 μg/kg estimated intake levels of formula-fed infants and 0.043–14.7 μg/kg in young children (NTP 2008); further, these doses have been reported to cause preneoplastic and neoplastic changes in perinatally exposed male (Ho et al. 2006) and female (Durando et al. 2007; Murray 2007; Newbold et al. 2007) experimental animal models. The present study adds to the growing body of literature that reports adverse effects following developmental exposure to low doses of BPA.

Among the benign abnormalities was an elevated incidence of ovarian cysts (67% in the BPA-1 group). Although ovarian cysts are histologically similar to those seen in our aged controls, the incidence is significantly higher than the controls in this study (25%) and in our historical controls; interestingly, the incidence is similar to that reported following neonatal BPA exposure (70%) (Newbold et al. 2007). The incidence following either pre natal or neonatal BPA is higher than we have observed in mice developmentally exposed to 0.001 mg/kg DES (58%) (Newbold et al. 1990), 50 mg/kg genistein (41%) (Newbold et al. 2001), or tamoxifen (60%) (Newbold et al. 1997), suggesting that the ovary may be a particularly sensitive target for the effects of BPA. Ongoing studies in our laboratory are investigating this possibility as well as mechanisms involved in the formation of ovarian cysts.

In one BPA-10 mouse, we observed prominent paraovarian cysts of mesonephric (Wolffian) duct origin similar to those reported in neonatally exposed mice (Newbold et al. 2007). This lesion, combined with the finding of prominent cystic Wolffian duct remnants in the uterine wall of BPA mice, also suggests that the mesonephric duct system (Wolffian duct) may be a target of BPA because both cystic structures have the same fetal tissue origin. Mesonephric-derived tissues have been shown to be sensitive to the effects of perinatal DES exposure in both male (Newbold et al. 1985a) and female mice (Haney et al. 1986).

Another BPA-induced abnormality found in all dose groups is PPL of the oviduct. PPL has been described in mice perinatally treated with DES. DES was shown to interfere with the normal differentiation of the Mullerian duct (the precursor of the oviduct), resulting in structural (prenatal exposure) (Newbold et al. 1983) and cellular (neonatal exposure) alterations (Newbold et al. 1984, 1985b). The molecular mechanism likely involves altered *HOX* gene expression in the differentiation of the reproductive tract (Taylor et al. 1997) because prenatal DES delays the expression of these genes (Ma et al. 1998). Subsequent studies suggest that DES works through multiple gene pathways (Miller et al. 1998; Pavlova et al. 1994). Thus, molecular “misprogramming” is mostly likely responsible for DES, as well as BPA-induced, oviductal alterations. Whether these compounds cause these effects through the classical ER-α or ER-β, or the newly identified ncmER (Alonso-Magdalena et al. 2005) pathways, or some other pathway, remains to be determined.

The benign lesions (CEH and adenomyosis) also occurred in the uterus of mice prenatally treated with BPA, but the incidence was not statistically different from controls. Although these lesions were histologically similar to those in aged controls, the lesions were more severe and their involvement in the uterine horns was more extensive in the BPA-treated groups compared with controls in this study and with our historical controls.

Of particular significance in this study is the occurrence of more severe ovarian lesions (cystadenoma) in the three highest BPA dose groups. In the uterus, adenocarcinoma was not observed in this study, although atypical hyperplasia, its premalignant lesion, was present. We were not suprised by the lack of uterine adenocarcinoma because BPA has weak ER-α binding and because the induction of uterine epithelial tumors is associated with a chemical’s binding affinity for this particular receptor during neonatal life (Newbold and Liehr 2000; Newbold et al. 2006). In the uterus, we also found an increased incidence of stromal polyps in the BPA-100 group. These lesions are considered preneoplastic/neoplastic in experimental rodent models because they are often the site for the development of endometrial stromal sarcoma (Davis et al. 1999; Maronpot 1999). Historically, we have rarely seen stromal polyps in CD-1 mice, although we did observe this lesion in one control mouse in a previous study (Newbold et al. 2007). Interestingly, in the present study, we identified a large invasive stromal sarcoma of the cervix after prenatal BPA-100 exposure; we have never seen this malignant lesion in any of our historical controls. Taking the stromal polyps and the stromal sarcoma together, these lesions suggest that stromal tissue is a target for BPA exposure, especially if exposure occurs during critical periods of differentiation of the reproductive tract. A similar finding has been shown in mice exposed to low-dose DES (Newbold et al. 2002).

We identified adverse effects in the reproductive tract in all BPA-treated groups, but it is interesting that the lowest dose (BPA-0.1) was the most affected (Table 3). Non linear dose–response curves have been commonly reported in endocrinology studies (vom Saal et al. 1997). One explanation for these effects can be found in DNA microarray studies (Coser et al. 2003; Shioda et al. 2006), where increasing doses of estrogens, from low to higher levels, result in entirely different arrays of genes that are turned on or off. Thus, the idea that there should be only a quantitative change in end points as the dose increases is not supported by these studies; instead, entirely different types of effects could occur as the high dose range is reached (for example, the changes we observed in the BPA-1000 group in the present study). This requires further investigation; however, the pattern of nonmonotonic effects is similar to what we observed in our neonatal BPA study (Newbold et al. 2007).

In a previous NTP carcinogenesis bioassay, the NTP (1982) reported that adult exposure to BPA was associated with cancers of the hematopoietic system. In the present study, we found one BPA-exposed mouse (BPA-1; 7 months of age) had a poorly differentiated sarcoma that infiltrated one ovary and the entire reproductive tract; we consider this lesion to be hematopoietic in origin. Although this study was designed to address only long-term, mainly carcinogenic, effects of BPA on reproductive tissues, certainly, the effects on the hematopoietic system warrants further follow-up.

In the present study, body weights were not different between BPA-treated mice and control mice. This lack of difference is most likely due to the advanced age of the mice in the study. Our laboratory and others have previously reported that developmental exposure to BPA, DES, and other environmental chemicals with endocrine-disrupting effects is associated with obesity in mice after they reach puberty and throughout maturity (Grun and Blumberg 2006; Grun et al. 2006; Howdeshell et al. 1999; Miyawaki et al. 2007; Newbold et al. 2005, 2007; Rubin et al. 2001); however, the animals in those studies were not examined at 18 months of age. Also, we have shown that significant differences in body weight in DES-treated mice compared with controls at 6–8 months of age become more difficult to detect as the animals age because of increased individual variability among all mice and because of increased disease and tumors (Newbold et al. 2007); thus, this variability probably accounts for lack of detection of body weight differences in this study. More important, we found no apparent correlation of body weight and tumor occurrence in either individual animals or groups, although a few BPA-exposed mice (but no controls) died before the completion of the study.

In summary, the findings of the present study raise concerns about widespread exposure to BPA and, in particular, exposure to fetuses, infants, and children. *In vitro* studies showing that BPA transforms SHE cells (Tsutsui et al. 2000) and induces aneuploidy (Tsutsui et al. 1998), and previous *in vivo* studies showing that BPA causes mammary tumors (Durando et al. 2007; Murray 2007) and preneoplastic prostatic lesions (Ho et al. 2006), along with evidence of BPA carcinogenicity following adult exposure (Huff 2001) together indicate that the body of literature merits serious consideration. Although studies are needed to determine the potential adverse effects to humans exposed to BPA during critical stages of neo natal or early development, the potential risks and benefits should be thoroughly assessed to determine the appropriate balance of exposures of this chemical during development and the permanent effects that may follow.

## Figures and Tables

**Table 1:** Distribution of mice by treatment group.

Prenatal treatment	No. of treated dams	No. of pups at start	Died early but not necropsied (*n*)^a^	Died early and necropsied (*n*)	Total no.
Control	5	16	0	0	16
BPA-0.1	5	16	2	1^b^	14
BPA-1	5	16	3	1^c^	13
BPA-10	5	16	2	1^d^	14
BPA-100	5	16	2	0	14
BPA-1000	5	16	3	3^e^	13

a Not included in the study.

b At 16 months of age.

c At 7 months of age.

d At 8 months of age.

e Two at 8 months of age and one at 10 months of age.

**Table 2:** Incidence [fraction (%)] of abnormalities in the ovary/oviduct and uterus of aged mice treated pre natally with BPA.

Prenatal treatment	Ovary/oviduct	Uterus
Control (*n* = 16)	No CL, 1/16 (6) Ovarian cysts, 4/16 (25) PPL, 0/16 (0)	CEH, 2/16 (13) Adenomyosis, 1/16 (6)

BPA-0.1 (*n* = 14)	No CL, 2/14 (14) Ovarian cysts, 4/14 (29) PPL, 1/13^a^ (8)	CEH, 0/14 (0) Adenomyosis, 1/14 (7) Atypical hyperplasia, 3/14 (21) Stromal polyp, 1/14 (7) Prominent Wolffian remnants, 1/14 (7)

BPA-1 (*n* = 13)	No CL, 3/12^a^ (25) Ovarian cysts, 8/12^a^ (67)* PPL, 1/12^a^ (8)	CEH, 5/13 (38) Adenomatous hyperplasia, 1/13 (8) Atypical hyperplasia, 2/13 (15) Squamous metaplasia, 1/13 (8) Stromal polyp, 1/13 (8) Prominent Wolffian remnants, 1/13 (8)

BPA-10 (*n* = 14)	No CL, 1/14 (7) Ovarian cysts, 2/14 (14) Prominent paraovarian cysts, 1/14 (7) Ovarian cystadenoma, 1/14 (7) PPL, 3/13^a^ (23)	CEH, 1/14 (7) Adenomyosis, 2/14 (14) Stromal polyp, 1/14 (7) Prominent Wolffian remnants, 2/14 (14)

BPA-100 (*n* = 14)	No CL, 1/14 (7) Ovarian cysts, 5/14 (36) Ovarian cystadenoma, 1/14 (7) PPL, 3/13^a^ (23)	CEH, 5/14 (36) Squamous metaplasia, 2/14 (14) Adenomatous hyperplasia, 2/14 (14) Stromal sarcoma of cervix, 1/14 (7)

BPA-1000 (*n* = 13)	No CL, 1/13 (8) Ovarian cysts, 5/13 (38) Ovarian cystadenoma, 1/13 (8) PPL, 2/13 (15)	CEH, 1/13 (8) Hyperplasia, 2/13 (15) Hypoplastic, 1/13 (8) Atypical hyperplasia, 1/13 (8) Prominent Wolffian remnants, 2/13 (15)

Abbreviations: CEH, cystic endometrial hyperplasia; CL, corpora lutea; PPL, progressive proliferative lesion of the oviduct.

a Appropriate tissue sections of the ovary/oviduct were not available for one mouse in the treatment group.

* *p* < 0.05 compared with the control group, by Fisher’s exact test.

**Table 3:** Summary of preneoplastic and neoplastic lesions [fraction (%)] in the ovary/oviduct and reproductive tract of aged mice treated prenatally with BPA.

	Ovary/oviduct		Reproductive tract		Total no. with lesions	
Prenatal treatment	Incidence of lesions	*p*-Value^a^	Incidence of lesions	*p*-Value^a^	Incidence of lesions	*p*-Value^a^
Control (*n* = 16)	0/16 (0)	—	0/16 (0)	—	0/16 (0)	—
BPA-0.1 (*n* = 14)	0/14 (0)	—	4/14 (29)	0.04	5/14 (36)^b^	0.01
BPA-1 (*n* = 13)	0/13 (0)	—	3/13 (23)	0.08	4/13 (31)^c^	0.03
BPA-10 (*n* = 14)	1/14 (7)	0.47	1/14 (7)	0.47	2/14 (14)	0.21
BPA-100 (*n* = 14)	1/14 (7)	0.47	1/14 (7)	0.47	2/14 (14)	0.21
BPA-1000 (*n* = 13)	1/13 (8)	0.45	1/13 (8)	0.45	2/13 (15)	0.19

Incidence does not include ovarian cysts or CEH.

a Relative to controls by Fisher’s exact test.

b Includes one animal with uterine hemangioma.

c Includes one animal with poorly differentiated sarcoma in the ovary and reproductive tract (probably hematopoetic in origin).
